# Effect of Temperature and Molarity on the Evaluation of Antimicrobial, Cytotoxic, and Antioxidant Activities of the Bio-Oil from Açaí Seed (*Euterpe oleracea* Mart.)

**DOI:** 10.3390/ijms26178251

**Published:** 2025-08-26

**Authors:** Iago Castro da Silva, Pamela Suelen da Silva Seabra, Kely Campos Navegantes Lima, Ricardo Barbosa Bezerra Filho, Alanna Lorena P. dos Santos, Amanda Caroline dos Santos Monteiro, Giovanna Quintero Pamplona, Alexandre Guilherme da Silva Dias, Rayane Caroline dos Santos Pereira, Leticia Araujo Costa, Thays Jhessica Mota Pinheiro, Lauro Henrique Hamoy Guerreiro, Nélio Teixeira Machado, Marta Chagas Monteiro

**Affiliations:** 1 Postgraduate Program in Pharmacology and Biochemistry, Faculty of Pharmacy, Federal University of Pará/UFPA, Belém 66075-110, PA, Brazil; castroiago95@gmail.com; 2 Postgraduate Program in Pharmaceutical Sciences, Faculty of Pharmacy, Federal University of Pará/UFPA, Belém 66075-110, PA, Brazil; pamela.seabra@ics.ufpa.br; 3 Faculty of Pharmacy, Campus Professional-UFPA, Federal University of Pará, Street Corrêa N° 1, Belém 66075-900, PA, Brazil; profkelynavelima@gmail.com; 4 Laboratory Immunology, Microbiology and In Vitro Assays (LABEIM), Faculty of Pharmacy, Federal University of Pará/UFPA, Belém 66075-110, PA, Brazil; ricardo.filho@ics.ufpa.br (R.B.B.F.); alannalorenapimentels@gmail.com (A.L.P.d.S.); amanda.monteiro@ics.ufpa.br (A.C.d.S.M.); giovanna.pamplona@ics.ufpa.br (G.Q.P.); alexandre.dias@ics.ufpa.br (A.G.d.S.D.); rayane.pereira@ics.ufpa.br (R.C.d.S.P.); 5 Faculty of Sanitary and Environmental Engineering, Campus Profissional-UFPA, Universidade Federal do 17 Pará, Rua Corrêa N° 1, Belém 66075-900, PA, Brazil; leticia.araujo.costa@itec.ufpa.br (L.A.C.); thaysjhessica7@gmail.com (T.J.M.P.); guerreirolauroengq@gmail.com (L.H.H.G.); machado@ufpa.br (N.T.M.)

**Keywords:** açaí, bio-oil, pyrolysis

## Abstract

Açaí (*Euterpe oleracea* Mart.), a fruit from the Amazon, is valuable both economically and nutritionally. Its seeds, often discarded, can be transformed into bio-oil through pyrolysis (a thermochemical degradation process of residual biomass), providing a sustainable alternative to fossil fuels. This study investigates how temperature and molarity influence the antimicrobial, antioxidant, and cytotoxic activities of the produced bio-oil. Various assays were performed on bio-oil samples obtained under different pyrolysis conditions—specifically, at temperatures of 350, 400, and 450 °C, and molarities of 0.5 M, 1.0 M, and 2.0 M—to evaluate antimicrobial, antioxidant, and cytotoxic activities. Gas chromatography–mass spectrometry (GC–MS) was used to analyze the composition, revealing that phenolic compounds were the most abundant (55.70%), followed by cyclic and aromatic hydrocarbons (11.89%), and linear hydrocarbons (9.64%). Despite a reduction in oxygenated compounds, the bio-oil maintained bacteriostatic activity against *Escherichia coli* and *Staphylococcus aureus*, especially at 350 °C. The antioxidant activity was highest at 350 °C and at lower molarities. Additionally, lower concentrations of acid impregnation showed cytotoxic effects at higher temperatures. Thus, bio-oil from açaí seeds produced via pyrolysis demonstrates potential for antioxidant and antimicrobial activities, suggesting viability for further testing at dilutions with lower cytotoxicity.

## 1. Introduction

The açaí (*Euterpe oleracea* Mart.) is a fruit typical of the Amazon region, belonging to the order Arecales and the family Arecaceae, valued for its nutritional properties and its role in the local economy [1,2,3]. The seeds, often discarded after pulp extraction, are an underutilized resource that can be harnessed through pyrolysis—a thermal process that transforms biomass into bio-oil, gas, and charcoal in the absence of oxygen. The bio-oil obtained from this process has great potential as a sustainable alternative to fossil fuels and can be used in various industrial and energy applications [4,5]. Among the various biomass sources, açaí seeds (*E*. *oleracea* Mart.) stand out as particularly promising, not only because of their abundance in the Amazon but also due to their rich chemical composition [1,4,6,7,8]. The study of the properties of bio-oil derived from these seeds opens new prospects for applications in various fields, especially in the pharmaceutical, food, and chemical industries [1,5,9,10,11,12,13,14,15].

Temperature and molarity are fundamental parameters that significantly influence the composition and properties of bio-oil. The pyrolysis temperature plays a crucial role in biomass degradation and the formation of desirable compounds. In turn, molarity affects the solubility and reactivity of the extracted compounds. Understanding how these factors interact is essential for optimizing the production and use of bio-oil, especially in applications that require specific biological activities. This understanding can open new opportunities for harnessing this resource across various industries [15,16,17,18,19,20,21,22].

The potential antimicrobial activities of bio-oil and other natural products derived from açaí biomass are highly relevant, especially in light of the growing issue of microbial resistance to conventional antibiotics. Studies show that natural compounds can provide an effective and less harmful alternative for treating infections. Therefore, investigating how temperature and molarity in the pyrolysis process influence these biological activities is crucial for developing new antimicrobial agents from natural sources such as açaí bio-oil [23,24,25,26,27,28].

In addition to its antimicrobial properties, the cytotoxic activities of bio-oil are equally important. Evaluating cytotoxicity is essential to ensure the safety of compounds for potential therapeutic use. The relationship between temperature, molarity, and cellular toxicity is an aspect that deserves further investigation, ensuring that bio-oil derivatives are not only effective but also safe for consumption [24,25]. Another important point is the antioxidant activity of bio-oil. Given the increasing attention to antioxidant compounds and their health benefits, it is essential to understand how temperature and molarity influence this activity. Antioxidant compounds play a significant role in the prevention of chronic diseases, and effective extraction is crucial to maximize the benefits of bio-oil [5,22,29].

Based on these principles, this article aimed to investigate in detail the effects of temperature and molarity on the antimicrobial, cytotoxic, and antioxidant activities of bio-oil extracted from açaí seeds. Through comprehensive analysis, it sought to contribute to the understanding of the interactions between these parameters, providing valuable information that can assist in the development of innovative and sustainable products in the pharmaceutical field.

## 2. Results and Discussion

### 2.1. Chemical Composition of Bio-Oil by Gas Chromatography–Mass Spectrometry (GC–MS)

The substances identified in the GC–MS are listed in Table 1. The precise identification of bio-oil components via GC–MS poses challenges, particularly due to the complex nature of the molecules and organic matrices involved. These studies underscore not only the diversity in bio-oil composition but also the significant influence of variables, such as temperature, ion presence, and biomass characteristics, on the formation and chemical structure of these pyrolysis-derived products. In this study, phenolic compounds stand out as the major components of the bio-oil, representing 55.70%, followed by cyclic and aromatic hydrocarbons (11.89%), and linear hydrocarbons (9.64%). These findings align with those reported by Sousa et al. [30], who identified the following major compounds in the pyrolyzed bio-oils of acerola seeds at 550 °C and 650 °C, in descending order: 9-octadecenoic acid; palmitic acid; linoleic acid; phenol; and *p*-cresol.

Similar studies, such as those by Gois et al. [30], indicate that the composition of the aqueous fractions from pyrolysis includes a mixture of compounds, including ketones, acids, phenols, nitrogenous compounds, and alcohols, with phenols being the predominant class. The phenolic area percentages varied from 25% (bean pods) to nearly 70% (grape seeds) compared to other classes. The aqueous fractions from pine nut shells, guava seeds, and grape seeds showed the highest percentages, exceeding 50%. This outcome can be attributed to the higher lignin content relative to the holocellulose content in the biomass, a pattern also observed in the bio-oil from açaí. In the specific context of açaí bio-oil, lignin forms a complex network of phenylpropanoid units during pyrolysis, with depolymerization of the cross-linked chains occurring at carbon–carbon and aryl–ether linkages.

Table 2 presents the chemical composition in area (%) of the GC–MS chromatogram for both liquid phases (aqueous and organic) of the bio-oil obtained from the pyrolysis of KOH 2.0 M-activated açaí seeds at 450 °C. As evidenced by Mahadevan et al. [31] and other authors, levoglucosan was not detected in the bio-oil or in the aqueous phase resulting from KOH-activated pyrolysis. Additionally, there was a significant reduction in the amount of carboxylic acids in the bio-oil, which decreased from 8.53% to 0.97%. According to these authors, an increase in carboxylic acids, such as acetic acid, was expected in the liquid fraction [31,32,33]. Chen et al. [34] also did not find acetic acid and suggested that the acid-base neutralization of the alkaline additive could be the reason for this result.

On the other hand, there was an increase in the amount of ketone compounds, rising from 3.53% to 7.07%. Mahadevan et al. [31] noted that the concentration of acetic acid can be influenced by the ketonization reactions of carboxylic acids, leading to the formation of ketones. These ketones can, in turn, undergo deoxygenation into hydrocarbons through ketonic decarboxylation, contributing to the presence of hydrocarbons in the bio-oil [35].

#### Effect of Temperature on the Chemical Composition of the Products

Table 3 presents the chemical composition and acidity of the aqueous phase generated from açaí seed pyrolysis. Chemical components and acidity were determined using GC–MS analysis. Detailed information on chemical functionalities, sum of peak areas, CAS numbers, and retention times for most of the identified compounds can be found in similar studies reported in the literature [5,19,22,36].

The results show a significantly higher concentration of phenolic compounds at lower pyrolysis temperatures (350 °C), indicating that temperature is a variable that influences the reduction in phenolic compounds and the increase in hydrocarbons in the solution. This can be observed in the studies conducted by De Castro et al. [36] and Valois et al. [5]. In addition, Silva et al. [37], demonstrates a significant presence of long-chain acids and phenolic compounds, attributed to the thermal cracking process during acerola residue pyrolysis. These phenolic compounds are heavily influenced by the pyrolysis temperature, which promotes secondary thermal degradation reactions that result in the transformation of these components into lower-molecular-weight substances.

Therefore, the precise identification of bio-oil components via GC–MS poses challenges, particularly due to the complex nature of the molecules and organic matrices involved. These studies underscore not only the diversity in bio-oil composition but also the significant influence of variables, such as temperature, ion presence, and biomass characteristics, on the formation and chemical structure of these pyrolysis-derived products. Phenolic compounds, in addition to their antioxidant functions, are associated with various health benefits such as anti-inflammatory, anticancer, and cardioprotective properties [5,38,39]. These additional benefits make these compounds highly valued not only for basic nutrition but also as essential components in healthy diets and medical therapies.

During pyrolysis, temperature is a crucial parameter that determines both the quantity and quality of compounds present in bio-oil. Understanding the impact of temperature allows for an optimization of the production of compounds like phenolics and hydrocarbons, ensuring that bio-oil meets specific requirements in diverse industries, from biofuels to high-value chemicals in the food, cosmetic, and pharmaceutical sectors [40,41].

The thermochemical degradation of biomass generally occurs at higher temperatures, leading to the decomposition of a greater amount of compounds in bio-oil, including phenolic compounds. In contrast, lower temperatures not only promote the formation of these compounds but also help to preserve them in bio-oil [15]. These results are consistent with previous studies demonstrating that increased temperatures reduce phenolic compounds [36,42].

The transformation of waste into value-added products reduces the amount of waste generated and minimizes the environmental impacts of improper disposal, such as river pollution, as well as the environmental wear and tear of landfills, requiring lower maintenance costs and fewer restoration actions [43]. Pyrolysis generates fewer pollutants and produces reusable products with significant energy or economic value. It is therefore a strategic mechanism for the circular bioeconomy and a renewable energy source [44]. The conversion of waste is dictated by the pyrolysis method used, which influences the economic viability of the process. At low temperatures, fast pyrolysis consumes less energy and heating time [45], which is important for forming bioproducts with potential biological activity.

### 2.2. Evaluation of the Antioxidant Potential of the Bio-Oil

#### 2.2.1. Effect of Temperature on the Antioxidant Activity of the Bio-Oil Using the TEAC Method

**Figure 1:**
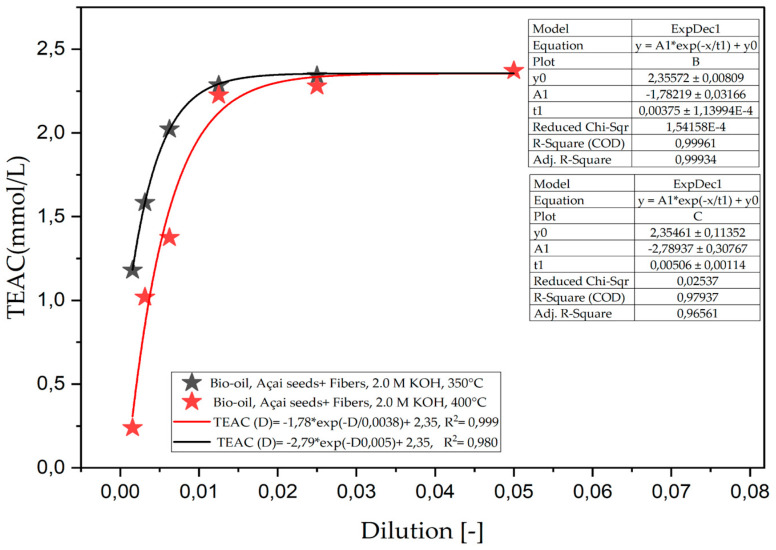
Total antioxidant capacity (TEAC) of the organic phase (bio-oil) obtained via the pyrolysis of açaí seeds + fiber, chemically activated with 2.0 M KOH solutions at temperatures of 350 and 400 °C on a laboratory scale.

Figure 1 presents the total antioxidant capacity (TEAC) of the organic phase of bio-oil obtained by the pyrolysis of açaí seeds and fiber, chemically activated with 2.0 M KOH solutions at temperatures of 350 and 400 °C on a laboratory scale. The results show that the total antioxidant capacity, measured by the TEAC method, decreases with increasing dilution, following a first-order exponential decay pattern, and presenting a coefficient of determination (R^2^) of 0.99 for 350 °C and 0.98 for 400 °C. This similarity indicates that, within this range, temperature was not a determining factor in modifying the antioxidant capacity of the samples.

These results are linked to the composition of the bio-oil, which can vary depending on the conditions under which the pyrolysis is carried out [46]. Despite this, at a low dilution, there were no significant variations between the temperatures of 350 °C and 400 °C, as shown in Table 4, showing that the antioxidant compounds present in the samples did not suffer from major degradation or volatilization during the pyrolysis process. However, from the 1:80 dilution onwards, the total antioxidant capacity in terms equivalent to Trolox became evident, with the loss of this small portion. According to the study by Valois et al. [22], which evaluated the concentration of oxygenates in relation to the temperature used in pyrolytic processing, there was a drop from 50.07 to 45.12 (area%) of phenols, 16.66 to 10.76 (area%) of ketones and 5.41 to 1.82 (area%) in bio-oil; the fraction lost in this process could be a determining factor in the drop in capacity mentioned in our results.

**Figure 2:**
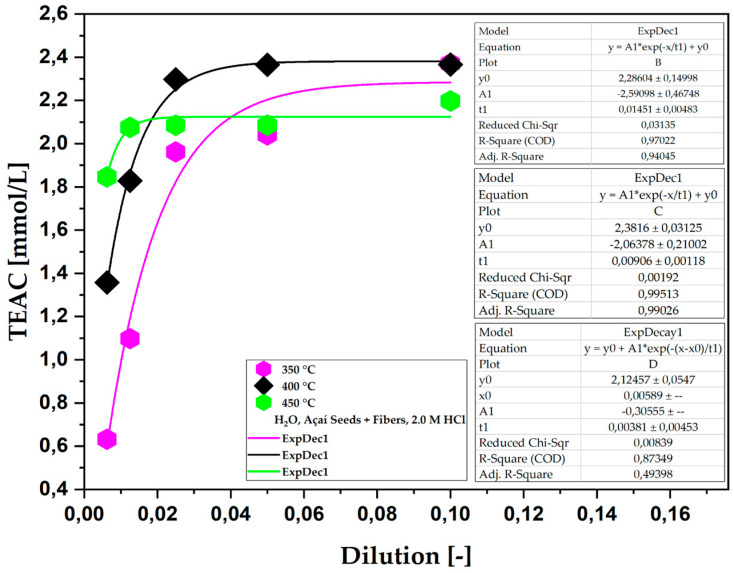
TEAC of the aqueous phase obtained via the pyrolysis of açaí seeds + fiber, chemically activated with 2.0 M HCl solutions at temperatures of 350, 400, and 450 °C on a laboratory scale.

Figure 2 shows the total antioxidant capacity (TEAC) of the aqueous phase obtained from the pyrolysis of açaí seeds + fibers, chemically activated with 2.0 M HCl solutions at temperatures of 350, 400, and 450 °C on a laboratory scale. The experiments show that the total antioxidant capacity, measured by the TEAC method, decreases as the dilution increases, following a first-order exponential decay pattern. It was also observed that, from a dilution of 1:40, the total antioxidant capacity in Trolox equivalents decreased considerably, indicating that, for this dilution ratio, the antioxidant capacity remained practically constant.

According to the results shown in Table 5, it is possible to understand that increasing the pyrolysis temperature from 350 °C to 450 °C does not cause a significant variation in the total antioxidant capacity (TEAC) of the bio-oils. This difference indicates that, although pyrolysis at different temperatures can affect the chemical structure of the biomass and the formation of antioxidant compounds, the effect of temperature in this range (350 °C to 450 °C) is considerably low, indicating that in the samples impregnated with HCl, the phenolic compounds and other antioxidants formed during pyrolysis have good thermal resistance and are thus able to maintain their antioxidant function even at higher temperatures. 

#### 2.2.2. Effect of Molar Concentration on the Antioxidant Activity of Bio-Oil Using the TEAC Method

**Figure 3:**
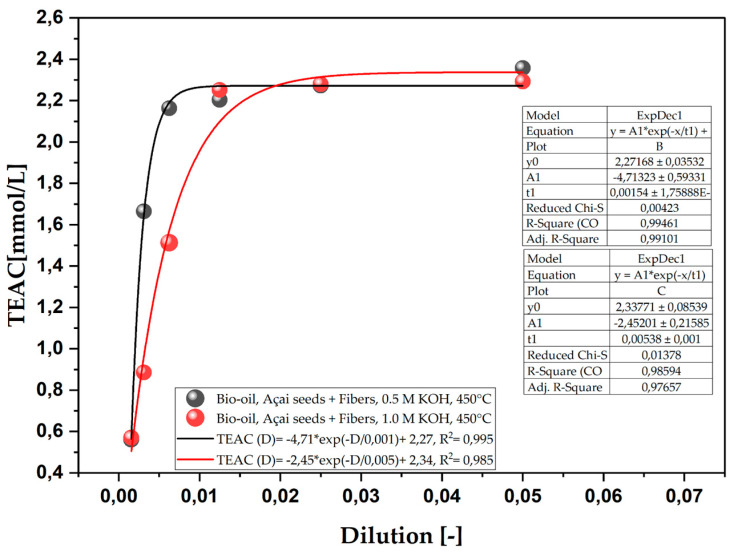
Total antioxidant capacity (TEAC) of the organic phase (bio-oil) obtained via the pyrolysis of açaí seeds + fiber, chemically activated with 2.0 M KOH solutions at a temperature of 450 °C on a laboratory scale.

The antioxidant capacities of bio-oils obtained by the pyrolysis of açaí seeds (*E. oleracea*) using different concentrations of KOH (0.5 and 1.0 M) at a temperature of 450 °C and atmospheric pressure, under laboratory conditions, are shown in Figure 3. A regression analysis shows that both models present an excellent fit (R^2^ of 0.995 for 0.5 M and 0.985 for 1.0 M), demonstrating that the relationship between dilution and antioxidant capacity follows a first-order decay behavior. 

The difference observed between the 0.5 M and 1.0 M concentrations of KOH, although small, suggests that the concentration of KOH influences the amount of antioxidant compounds released during pyrolysis. The fact that the 1.0 M solution has a higher antioxidant capacity indicates that a higher degree of chemical activation results in a more efficient release or preservation of antioxidant compounds in the bio-oil. This behavior can be attributed to KOH’s greater ability to break lignocellulosic bonds or modify the organic matrix, facilitating the formation or release of phenolic compounds, known for their high antioxidant activity [47].

Table 6 shows the antioxidant capacity of the bio-oils obtained from the pyrolysis of açaí seeds (*E. oleracea*), chemically activated with KOH solutions at concentrations of 0.5 M and 1.0 M at 450 °C. By analyzing the results, it is possible to infer that increasing the molarity resulted in a drop in the oxidizing capacity. This observation can be explained by the possible saturation of the active sites of KOH at higher concentrations, which can lead to non-interaction with antioxidant compounds or the formation of products that do not add to the antioxidant capacity [48]. This effect suggests that the ideal concentration of KOH to maximize antioxidant activity may be around 0.5 M, since at 1.0 M, the drop in TEAC occurs more abruptly and earlier.

**Figure 4:**
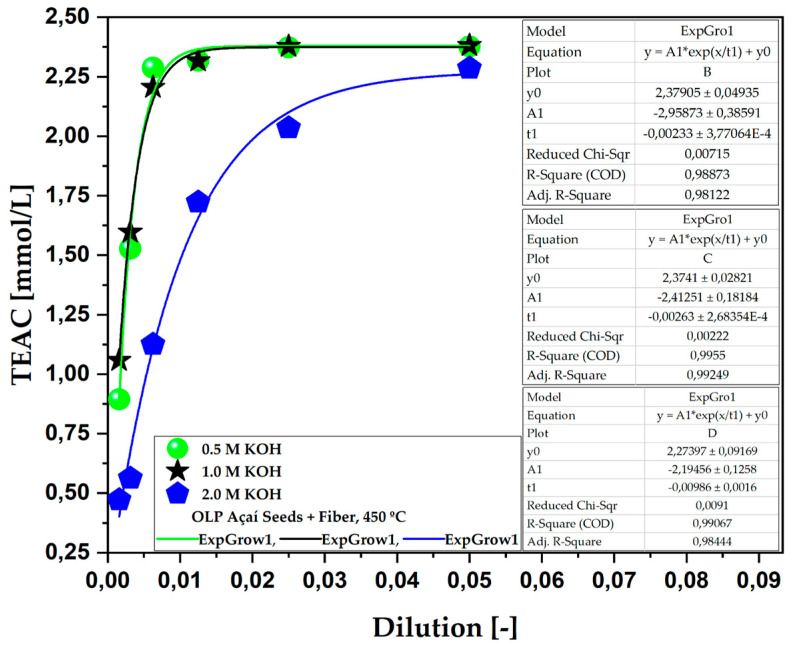
TEAC of the aqueous phase obtained via the pyrolysis of açaí seeds + fiber, chemically activated with KOH solutions at 450 °C and molarities of 0.5 M, 1.0 M, and 2.0 M on a laboratory scale.

Figure 4 and Table 7 show the results of the total antioxidant capacity of bio-oils obtained from the pyrolysis of açaí seeds, activated with different molarities of KOH at 450 °C. The experimental results clearly show that bio-oils obtained from the pyrolysis with 0.5 and 1.0 M KOH solutions at 450 °C exhibited greater antioxidant activity compared to those produced with a 2.0 M KOH solution. 

At higher concentrations of KOH, such as 2.0 M, the reaction can promote excess oxidation, degradation or even the non-activation of mechanisms that result in obtaining bioactive compounds that contribute to antioxidant capacity, since not all the reactions promoted by pyrolysis have been elucidated and it is a very complex process. With regard to the 0.5 M and 1.0 M molarities, both had practically identical results, and did not exceed the value of the other; therefore, it can be concluded that the results reached the plateau of the method applied.

#### 2.2.3. Effect of Temperature on Antioxidant Activity of Bio-Oil Using the DPPH• Method

**Figure 5:**
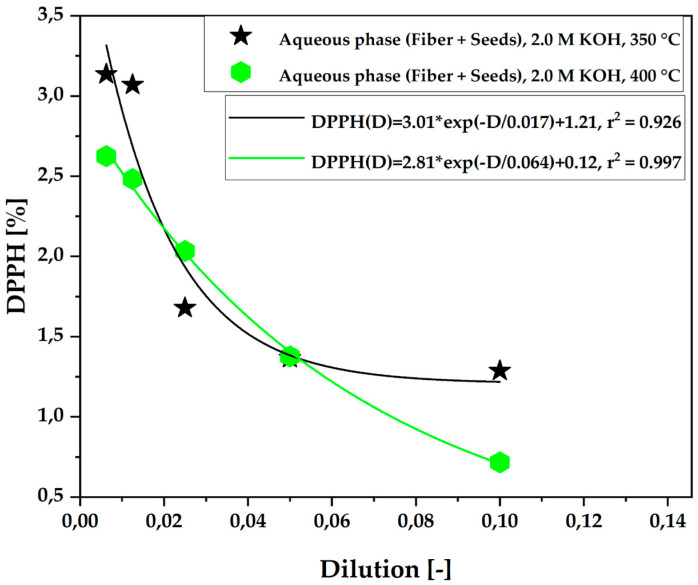
Antioxidant capacity by the (DPPH•) method of the aqueous phase obtained via pyrolysis of açaí seeds + fiber, chemically activated with 2.0 M potassium hydroxide (KOH) solutions at temperatures of 350 °C and 400 °C.

Figure 5 illustrates the antioxidant capacity (DPPH•) of the aqueous phase obtained from the pyrolysis of açaí seeds and fiber, chemically activated with 2.0 M solutions, at temperatures of 350 °C and 400 °C on a laboratory scale. The experiments reveal that the total antioxidant capacity, measured by the DPPH• method, increases as the capture of the DPPH• radical in the sample increases, reducing its concentration, following a pattern of first-order exponential decay. Additionally, it was observed that the temperature of 400 °C favored greater capture of the DPPH• radical, as shown in Figure 5. At this temperature, the radical concentration in the solution decreases with an increase in antioxidant capacity at a 1:10 dilution. Although the antioxidant capacity increased, its variation compared to the 350 °C temperature was minimal, indicating that, for this dilution ratio, antioxidant capacity remains nearly constant.

As shown in Table 8, an increase in antioxidant activity can be observed as radical capture increases. At 350 °C, the antioxidant capacity remained constant at lower dilutions (more concentrated samples), specifically at ratios of 1:10 and 1:20. In contrast, at 400 °C, there was higher radical capture only at the 1:10 dilution, indicating that more concentrated samples exhibited higher antioxidant activity in this temperature range. These findings are consistent with those in previous studies by De Castro et al. [36] and Valois et al. [5], which demonstrate that increasing the temperature promotes a reduction in phenolic compounds.

**Figure 6:**
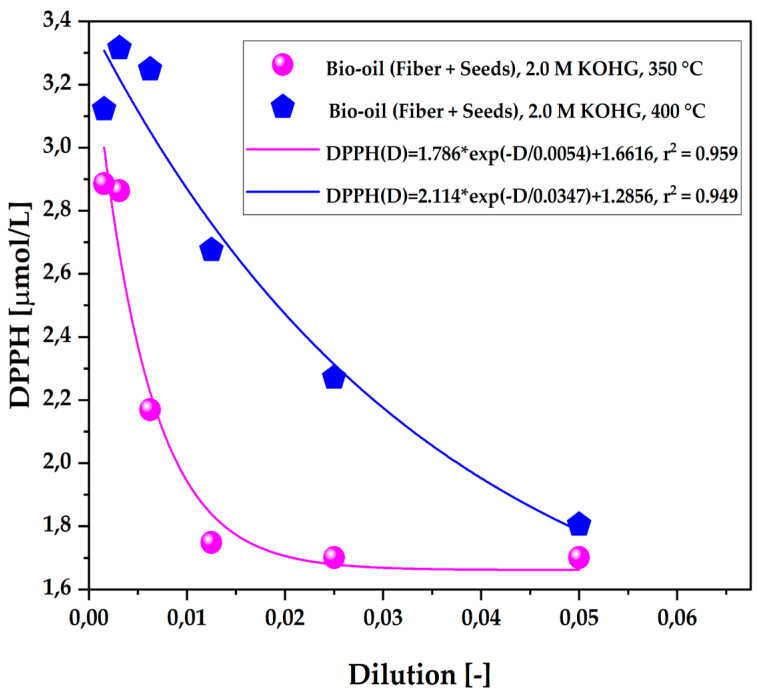
Antioxidant capacity by the 1,1-diphenyl-2-picrylhydrazyl (DPPH•) method of the organic fraction of bio-oil obtained via pyrolysis of açaí seeds + fiber, chemically activated with 2.0 M KOH solutions at temperatures of 350 °C and 400 °C on a laboratory scale.

The Figure 6 illustrates the antioxidant capacity of the organic fraction of bio-oil obtained from the pyrolysis of açaí seeds and fiber, activated with 2.0 M KOH solutions, at temperatures of 350 °C and 400 °C on a laboratory scale. According to experimental results, the total antioxidant capacity, measured by the DPPH• method, increases as the concentration of DPPH• radicals in the sample decreases, following a first-order exponential decay pattern. The pyrolysis temperature proved crucial for this antioxidant activity, with significantly more efficient radical capture observed at 350 °C. In this thermal range, there was a substantial reduction in radicals, indicating high efficacy in antioxidant capacity.

As shown in Table 9, there was an increase in antioxidant activity as the scavenging of free radicals increased, resulting in a decrease of the compound in the samples. This pattern was consistent at higher concentrations, particularly in the less diluted samples treated at 350 °C. Radical scavenging and the increase in antioxidant capacity were more pronounced at 350 °C compared to 400 °C. These findings highlight significant antioxidant activity across all dilutions of the aqueous fraction samples, with a particular emphasis on the less diluted samples at 350 °C.

Similar results were observed in the study by Oliveira et al. [49], which analyzed the antioxidant activity of essential oils from different genotypes, along with their major compounds. The oils demonstrated antioxidant capacity using the DPPH free radical scavenging method. Essential oils from *C. grewioides*, CGR112 and CGR104, exhibited moderate activity, with 23.66% and 18.65% radical scavenging, respectively. On the other hand, essential oil CGR106 showed the highest radical scavenging activity, reaching 67.91%, followed by essential oil CGR126 with 61.98%. It was also noted that the major compound eugenol, a phenolic monoterpene, demonstrated higher activity compared to other tested compounds.

#### 2.2.4. Effect of Molar Concentration on the Antioxidant Activity of Bio-Oil Using the DPPH Method

**Figure 7:**
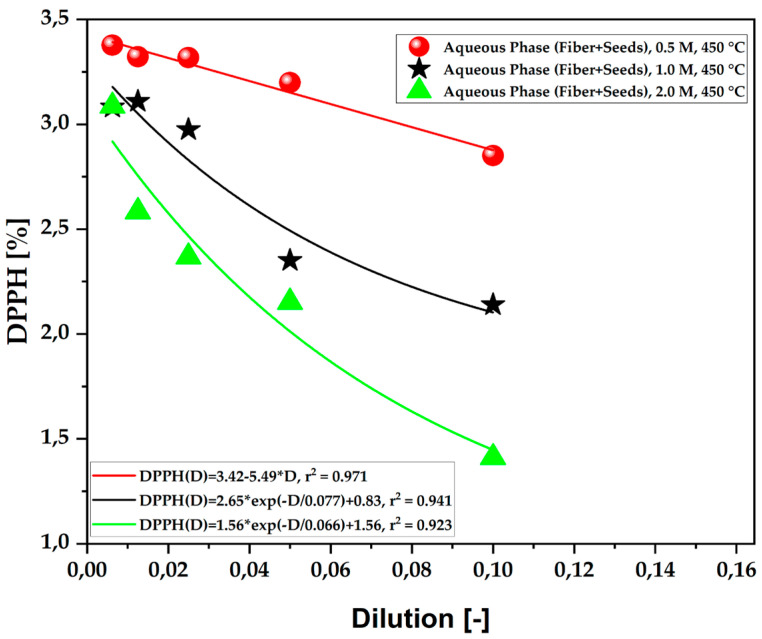
Antioxidant capacity by the 1,1-diphenyl-2-picrylhydrazyl (DPPH•) method of the aqueous fraction of bio-oil obtained via the pyrolysis of açaí seeds + fiber, chemically activated with KOH solutions at different molarities (0.5 M–2.0 M) at a temperature of 450 °C on a laboratory scale.

Table 10 presents the antioxidant capacity results of the aqueous fraction of bio-oils obtained from the pyrolysis of açaí seeds (*E. oleracea*), using different concentrations of KOH ranging from 0.5 to 2.0 M. The experiment was conducted at a temperature of 450 °C under atmospheric pressure in a laboratory setting. The data obtained are graphically illustrated in Figure 7. The results demonstrate significant antioxidant capacity for all tested KOH concentrations following a first-order exponential decay model. Notably, the 2.0 M concentration stood out among the more diluted samples, showing remarkable free radical scavenging capacity, resulting in extremely high inhibition at dilutions of 1:10 and 1:20.

**Figure 8:**
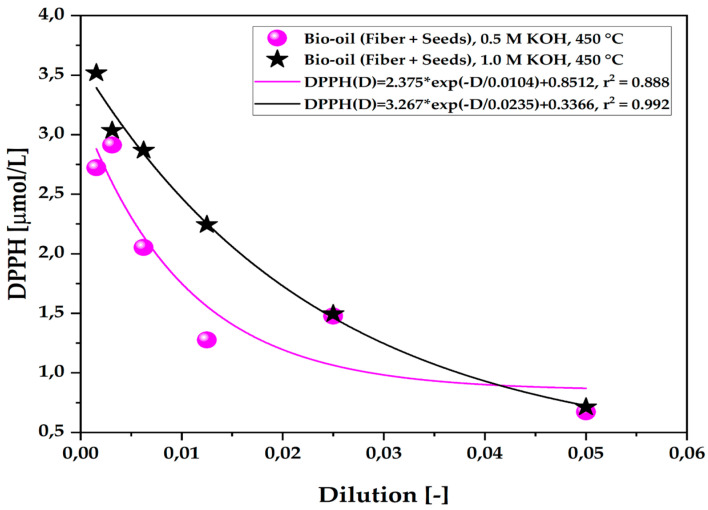
Antioxidant capacity by the 1,1-diphenyl-2-picrylhydrazyl (DPPH•) method of the organic fraction of bio-oil obtained via the pyrolysis of açaí seeds + fiber, chemically activated with KOH solutions at different molarities (0.5 M–2.0 M) at a temperature of 450 °C on a laboratory scale.

The data on the organic fraction of the bio-oil show similar results to the previously mentioned studies. As illustrated in Figure 8, the antioxidant capacity of bio-oils produced from açaí seeds (*E. oleracea*) via pyrolysis was analyzed using different concentrations of KOH (0.5 M and 1.0 M) at a temperature of 450 °C and atmospheric pressure on a laboratory scale. The results presented in Table 11 demonstrate that bio-oils obtained with KOH solutions at concentrations of 0.5 M and 1.0 M at 450 °C showed higher antioxidant activity at dilutions of 1:10 and 1:20, with a notable emphasis on the 1.0 M concentration. A first-order exponential decay was observed, indicating a lower number of free radicals in the samples, suggesting a high antioxidant potential and greater radical scavenging capacity.

Different temperatures generate intermediate reactions, leading to the formation of various chemical compounds in bio-oil, alongside gaseous and solid phases with specific characteristics [19]. The TEAC and DPPH methods demonstrated significant antioxidant activity of bio-oil (organic fraction) at various molarities, both with KOH and HCl, at 450 °C, in dilutions ranging from 1:80 to 1:160. This antioxidant activity can be attributed to the high concentration of oxygenated compounds, especially phenolics, present at this temperature.

Recent studies show that high temperatures (450 °C) during the pyrolysis of activated açaí seeds with a 2.0 M KOH solution do not favor the production of oxygenated compounds in bio-oil, resulting in the predominance of hydrocarbons. In contrast, lower temperatures (350 °C) promote the formation of oxygenated compounds, especially phenolics, in the bio-oil of these seeds, which are strongly associated with high antioxidant activity [22]. In summary, the thermochemical degradation of biomass suggests that high temperatures lead to the loss of valuable compounds, such as phenolics, in bio-oil. On the other hand, lower temperatures not only promote their formation but also help to preserve these compounds [22]. Thus, bio-oil stands out as a product rich in important chemicals that are especially relevant to the food, cosmetic, and pharmaceutical industries.

Phenolic compounds play a crucial role in bio-oil and have garnered significant interest due to their potential as natural sources of antioxidants, nutraceuticals, and preservatives in the food industry [41]. With significant antioxidant properties, these compounds help to neutralize free radicals and reduce oxidative stress, inhibiting the oxidation of DNA, proteins, and lipids [39]. Furthermore, they offer various health benefits, including anti-inflammatory, anticancer, and cardioprotective properties [50].

### 2.3. Evaluation of the Antimicrobial Activity of Bio-Oil

#### 2.3.1. Activity against *Escherichia coli*

Studies in the literature have indicated a growing concern regarding antibiotic resistance among bacteria, especially *E. coli*, a major cause of bacterial infections in humans, particularly Gram-negative bacterial infections [42]. Currently, strains carrying extended spectrum β-lactamases (ESBLs) are highlighted for their resistance to third-generation cephalosporins. Additionally, these bacteria have shown resistance to fluoroquinolones and gentamicin [51]. In light of this concerning scenario, the potential antimicrobial activity of açaí seed bio-oil against these bacteria was evaluated.

Based on information regarding *E. coli* strain ATCC 25922, various dilutions of the bio-oil were examined for their antimicrobial activity. The results of the minimum inhibitory concentration (MIC) and minimum bactericidal concentration (MBC) in relation to samples of wash water prior to the pyrolysis process are presented in Table 12.

Table 12 shows that in the wash water samples (L1, L2, and L3), the lowest concentration with a bacteriostatic effect for impregnation with 0.5 M KOH falls within a dilution range greater than 1:10. This pattern differs for higher molarities (1.0 M and 2.0 M), where dilutions for MIC and MBC were observed to be 1:40 for both. These results indicate that the bacteriostatic effect of the wash samples on the *E. coli* strain increases with the higher molarities used in the chemical pretreatment of lignocellulosic biomass from açaí seeds. The consistency of these effects suggests that, even at more diluted concentrations, these groups maintained bacteriostatic capability.

Table 13 presents the various dilutions of açaí seed bio-oil (aqueous phase) evaluated for their antimicrobial activity against *E. coli*, displaying the results of MIC and MBC. In the analysis of samples from the aqueous fraction of bio-oil impregnated with KOH at 450 °C, it can be observed that the lowest concentration exhibiting a bacteriostatic effect in the impregnation with 0.5 M KOH lies within a dilution range of 1:40. These results vary for higher molarities (1.0 M and 2.0 M), where dilutions for MIC and MBC of 1:20 and 1:10, respectively, were observed. These data indicate that the bacteriostatic effect of the aqueous fraction of bio-oil on *E. coli* strain decreases as the molarity used in the chemical pretreatment of lignocellulosic biomass from the açaí seeds increases at 450 °C [5]. However, even with the reduction in oxygenated compounds, especially phenolics, in the samples, the groups maintained their bacteriostatic capacity due to the increased molarity at 450 °C.

Table 14 presents the various dilutions of açaí seed bio-oil (organic fraction) evaluated for their antimicrobial activity against *E. coli*, showing the results of MIC and MBC. The results from samples of the organic fraction of bio-oil, subjected to impregnation with KOH under different temperature conditions (350 °C, 400 °C, and 450 °C) and molarities (0.5 M, 1.0 M, and 2.0 M), revealed that the concentration exhibiting bacteriostatic effects in impregnation with 0.5 M KOH falls within a dilution range of 1:80. However, for higher molarities (1.0 M and 2.0 M), the results showed different dilution values for MIC and MBC, namely, 1:40 and 1:80, respectively. These findings indicate that the bacteriostatic effect of the organic fraction of bio-oil, as well as the aqueous fraction, against *E. coli* strain increases with the higher molarity used in the chemical pretreatment of lignocellulosic biomass from the açaí seeds. Surprisingly, the results showed satisfactory efficacy even at lower concentrations, maintaining their bacteriostatic capacity despite the reduction in oxygenated compounds, especially phenolics, in the samples, due to the increased molarity and temperature during the pyrolysis process of lignocellulosic biomass [5,22].

**Figure 9:**
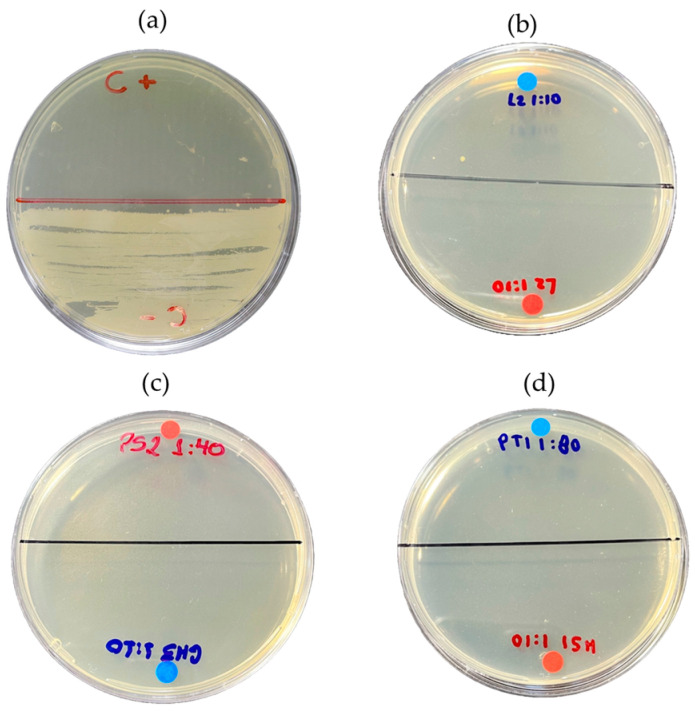
Minimum bactericidal concentration (MBC): (**a**) positive control (*E. coli*) and negative control (chloramphenicol 5 mg/mL); (**b**) MBC corresponding to wash water; (**c**) aqueous fraction; and (**d**) organic fraction against *E. coli* strain.

The Figure 9 shows the visual results of the MBC of the açaí wash water samples, aqueous fraction, and organic fraction of bio-oil against *E. coli* strain. As depicted in the image, no bacterial growth was observed within the ranges established in the results of this study.

The results of the antimicrobial activity of açaí bio-oils are supported by studies highlighting phenolic compounds derived from lignin as antimicrobial agents. In various investigations, bio-oil has been evaluated as an alternative wood preservative due to its ability to inhibit the growth of deteriorating microorganisms.

Studies on the antimicrobial effects of essential oils encompass a variety of research. For example, Puvaca et al. [52] analyzed the antimicrobial activity of essential oils from *Melaleuca alternifolia* and *Eucalyptus globulus* against the *E. coli* strain. The results showed that antimicrobial efficacy is associated with the composition of the main phytoconstituents of these oils. Specifically, *Melaleuca alternifolia* oil, with a high terpinen-4-ol content (38.53%), and *Eucalyptus globulus* oil, rich in 1,8-cineole (64.71%), were effective in reducing bacterial growth. These findings highlight that the properties of bioactive compounds present in essential oils play a crucial role in the observed antimicrobial activity, providing a solid scientific basis for exploring their therapeutic potential against bacterial infections such as those caused by *E. coli*.

#### 2.3.2. Activity against *Staphylococcus aureus*

The present study evaluated the antimicrobial capacity of açaí seed bio-oil at different temperatures and molarities against *S. aureus* strain ATCC 29213. The different dilutions of bio-oil were assessed for their antimicrobial activity; the results (MIC/MBC) concerning the samples of wash water prior to the açaí seed pyrolysis process are shown in Table 15.

Based on the results described in Table 15, it can be observed that both the MIC and MBC for impregnation with 0.5 M KOH fall within a dilution range of 1:5 and 1:20, respectively. These values differ for higher molarities (1.0 M and 2.0 M), where the dilution values for MIC and MBC are >1:10 and 1:40, respectively. As discussed earlier, temperature was a variable that was absent because the corresponding wash water samples did not undergo high-temperature pyrolysis.

These results suggest that the present study on the antimicrobial activity of wash samples against the *S. aureus* strain shows low MIC values, indicating high antimicrobial activity with dilutions greater than 1:5 (more concentrated samples), in accordance with the decrease in KOH treatment molarity.

The values of MIC and MBC of products from different dilutions of açaí seed bio-oil (aqueous phase) tested against the strain of *S. aureus* are presented in Table 16.

Based on the data from Table 16, it is observed that in the impregnation with KOH at a constant molarity of 2.0 M and at varying temperatures (350 °C, 400 °C, and 450 °C), both the MIC and MBC fall within a dilution range of 1:4. The molarities did not affect the results of the aqueous fraction samples as they were constant and equivalent to 2.0 M. Temperature, therefore, emerges as the determining variable in the concentrations of compounds present in the samples.

The results concerning the organic fraction samples of bio-oil can be seen in Table 17. The impregnation data with HCL at different temperatures (400 °C and 450 °C) and constant molarities equivalent to 2.0 M show that molarity did not alter the results, as it remained constant across samples. The determining factor is temperature, where at 450 °C, the MIC and MBC values were observed at higher dilutions, indicating less concentrated samples. As discussed earlier, increasing temperatures led to a reduction in oxygenated compounds and consequently diminished the antimicrobial capacity of the samples.

**Figure 10:**
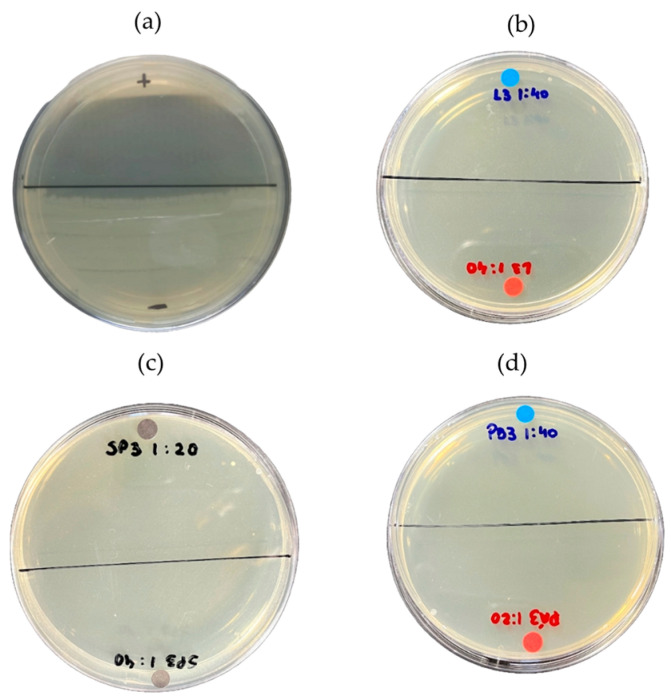
Minimum bactericidal concentration (MBC): (**a**) positive control (*S. aureus*) and negative control (chloramphenicol 5 mg/mL); (**b**) MBC for wash water; (**c**) MBC for aqueous fraction; and (**d**) MBC for organic fraction against *S. aureus* strain.

Figure 10 shows the results of the MBC of wash water samples from açaí, the aqueous fraction, and the organic fraction of bio-oil against the strain of *S. aureus*. The image indicates that there was no bacterial growth in the areas specified by the findings of this study.

In the study by Ferenz et al. [53], *S. aureus* was sensitive to all investigated essential oils, with the lowest concentration showing inhibitory halo formation at 12.5% for *Cymbopogon flexuosus* essential oil. This study concluded that essential oils exhibited biological activity against *S. aureus*, particularly highlighting the potential antimicrobial activity of *C. flexuosus* oil against this bacterium.

The literature covers a wide range of studies investigating the antimicrobial effects of essential oils. For instance, Mashiba et al. [54] evaluated the antioxidant and antimicrobial activity of pyrolysis lignin fractions. The antimicrobial action of the samples showed similar results in inhibiting the growth of both *E. coli* and *S. aureus*. Pyrolysis lignin demonstrates antioxidant and antimicrobial properties, making it a promising material for use in smart packaging and pharmaceutical products [54].

### 2.4. Cell Viability Assay

**Figure 11:**
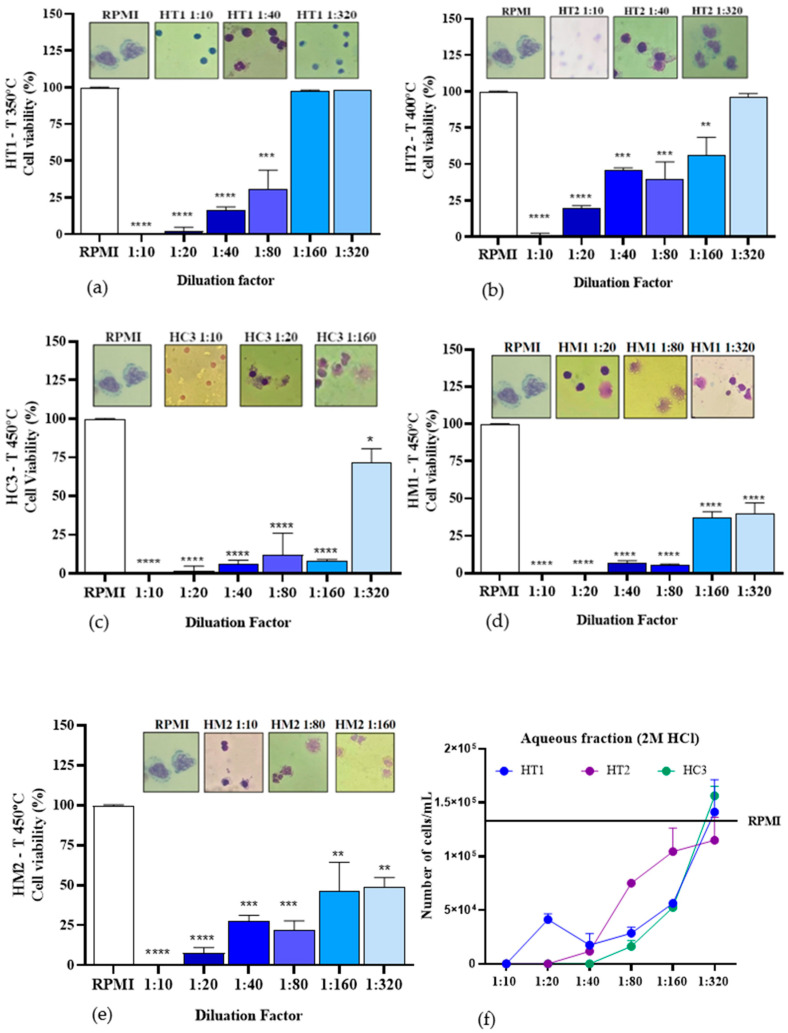
Percentage cell viability of PBMC after incubation with aqueous fractions impregnated with or without HCl at different temperatures and dilutions: (**a**) aqueous fraction impregnated with 2.0 M HCl at 350 °C; (**b**) aqueous fraction impregnated with 2.0 M HCl at 400 °C; (**c**) aqueous fraction impregnated with 2.0 M HCl at 450 °C; (**d**) aqueous fraction impregnated with 0.50 M HCl at 450 °C; (**e**) aqueous fraction impregnated with 1.0 M HCl at 450 °C; and (**f**) number of cells/mL in aqueous fractions HT1, HT2, and HC3. * *p* ≤ 0.05 ** *p* < 0.01 *** *p* < 0.00001.

Based on the results shown in Figure 11, at the beginning of the process, the cell viability of the aqueous fraction from the pyrolysis impregnated with 2.0 M hydrochloric acid (HCl) was investigated at the following different temperatures: 350 °C (HT1), 400 °C (HT2), and 450 °C (HT3). Increasing the pyrolysis temperature reduced cell viability at 1:160 dilutions (HT1, 97.5 ± 0.7%; HT2, 56.5 ± 12.0%; HT3, 8.5 ± 0.7%), but significantly improved cell viability at 1:320 dilutions (HT1, 98 ± 0%; HT2, 96.5 ± 2.1%; HT3, 71.5 ± 9.2%). Studies have shown that the aqueous fraction obtained by impregnation with 2.0 M HCl demonstrates a progressive increase in the acidity index as the pyrolysis temperature rises. This suggests that HCl-impregnated fractions contain a high quantity of oxygenated compounds, such as carboxylic acids and phenols, which consequently influence the acidity level [22]. At lower concentrations of acid impregnation (0.5 M-HM1; 1.0 M-HM2) and a pyrolysis temperature of 450 °C, a cytotoxic effect was observed at the tested dilutions, resulting in a reduction in both the number and appearance of viable cells compared to the 2.0 M HCl concentration at the same temperature (Figure 11d,e). This finding suggests that both the temperature and hydrochloric acid concentration significantly influence the components present in the aqueous fractions obtained from açaí pyrolysis. Since the acidity index is a crucial parameter for evaluating oil quality, its increase is related to the hydrolysis of triacylglycerols and the formation of free fatty acids, which can progressively increase with heating and exposure to light. This process can, in turn, elevate the cytotoxicity of the sample [55,56].

**Figure 12:**
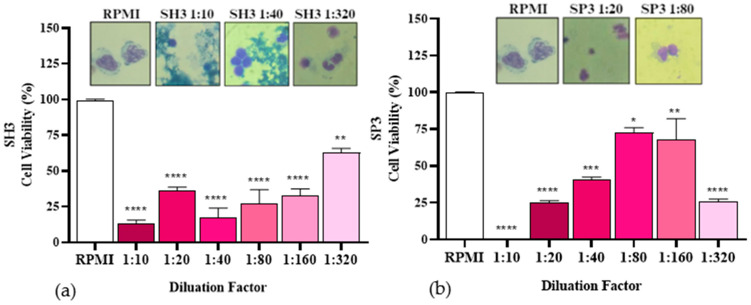
Cell viability in percentage of PBMC after incubation with the non-impregnated organic fraction obtained via pyrolysis at 450 °C. * *p* ≤ 0.05 ** *p* < 0.01 *** *p* < 0.00001 (**a**,**b**).

The results from the untreated aqueous solution at 450 °C (Figure 12a) demonstrated a lesser impact on cell viability compared to the samples HM1 and HM2, diluted at 1:320 (63 ± 2.8%), as discussed earlier. However, a reduction in cell survival was still observed.

The untreated organic fraction (SP3) at 450 °C showed lower cellular toxicity at dilutions of 1:80 (72.51 ± 3.5%) and 1:160 (68 ± 14.1%) (Figure 12b). Nevertheless, there was a decrease in cell viability compared to sample HT1, indicating that temperature influences the compounds extracted during pyrolysis, as discussed previously.

## 3. Materials and Methods

### 3.1. Bio-Oil Production

The bio-oil extracted from açaí seed residue was provided by the Laboratory of Sanitary and Environmental Engineering (LAESA) at the Federal University of Pará. The samples underwent different temperature processes at 350, 400, and 450 °C, as well as chemical pretreatment with potassium hydroxide (KOH). The samples obtained were divided into groups according to the product generated in the pyrolysis process and can be referred to as wash water, organic liquid product (PLO), and aqueous fraction [36].

### 3.2. Sample Preparation

#### 3.2.1. Wash Water

The wash water was obtained prior to the pyrolysis process by adding distilled water after the acidic or basic impregnation of açaí biomass, followed by filtration. After collecting the wash water, samples were prepared by diluting them with distilled water in the following ratios: 1:5 (200 µL of sample in 800 µL of distilled water); 1:10 (100 µL:900 µL); 1:20 (50 µL:950 µL); 1:40 (25 µL:975 µL); and 1:80 (12.5 µL:987.5 µL) [5].

#### 3.2.2. Aqueous Fraction

Considering their polar nature, the samples were diluted using only distilled water in ratios ranging from 1:5 to 1:80. The dilution of the aqueous fractions started at 1:5, where 200 µL of the sample was added to a 1.5 mL microcentrifuge tube (Kasvi) and then topped up to 1000 µL with 800 µL of distilled water. To prepare the dilutions of 1:10, 1:20, 1:40, and 1:80, 100 µL, 50 µL, 25 µL, and 12.5 µL of the sample were added to each microcentrifuge tube (KASVI, Pinhais, Brazil), respectively, and the volume was adjusted to 1000 µL by adding distilled water.

#### 3.2.3. Organic Fraction (PLO)

The samples of the organic fraction were categorized based on impregnation and temperature. Due to the non-polar nature of the sample, sample dilution (1:20 to 1:640) was performed according to a specific methodology. Initially, 800 µL of 10% dimethyl sulfoxide (DMSO-Merck, Darmstadt, Germany) in 1X PBS (ThermoFisher Scientific, Waltham, MA, USA) was added to a 1.5 mL microcentrifuge tube, followed by the addition of 200 µL of the sample to prepare the first dilution, which was then vortexed. Subsequent dilutions of 1:40, 1:80, 1:160, 1:320, and 1:640 were prepared for viability, antioxidant, and antimicrobial tests.

### 3.3. Identification of Components by Gas Chromatography (GC–MS)

Identification of components present in the oily samples from the pilot-scale experiments was performed using gas chromatography coupled with mass spectrometry (GC–MS). This analytical method involves volatilizing the sample and transporting it through a chromatographic column using a carrier gas (mobile phase) for separation. Chromatographic analyses were conducted on a chromatography system to separate and identify chemical compounds present in the bio-oils produced at semi-pilot and pilot scales. Component analysis was carried out using the Agilent Technologies system—Model CG-7890B coupled with a model MS5977A mass spectrometer, using a fused silica capillary column SLBTM-5 ms (30 m × 0.25 mm × 0.2).

### 3.4. Evaluation of the Antioxidant Potential of the Bio-Oil

#### 3.4.1. Evaluation of Antioxidant Activity Using the ABTS• + Radical Scavenging Method (TEAC)

The relationship of this activity to the reactivity of Trolox as a standard under identical conditions was calculated with the final results expressed in micromoles per liter (µM/L), corresponding to the concentration of Trolox with the antioxidant capacity equivalent to that of the sample under study. This measurement standard is referred to as TEAC (Trolox equivalent antioxidant capacity). The ABTS• + radical was prepared by reacting 5.0 mL of a solution containing 3840 μg mL^−1^ ABTS (Thermo scientific) with 88 μL of a solution containing 37,840 μg mL^−1^ potassium persulfate (ÊXODO CIENTIFICA^®^, Sumaré, Brazil), and the mixture was left in the dark for 16 h. After radical formation, the mixture was diluted in ethanol (MERCK^®^, Darmstadt, Germany) until an absorbance of 0.7 ± 0.01 at 734 nm was reached. For the OE concentrations (5 to 150 μg mL^−1^), reaction mixtures were prepared with the ABTS• + radical cation. In the dark, 30 μL aliquots of each OE concentration were withdrawn and transferred to test tubes containing 3.0 mL of the ABTS• + radical cation, then homogenized on a tube shaker (KASVI^®^ K45 2810, Pinhais, Brazil). After 6 min, the reaction mixture’s absorbance was measured using a spectrophotometer (GLOBAL TRADE TECHNOLOGY 190–1000 nm, São Paulo, Brazil) at 734 nm [57].

#### 3.4.2. Evaluation of Antioxidant Activity by the DPPH● Radical Scavenging Method

The total antioxidant activity was analyzed by assessing the ability of antioxidants present in the sample to neutralize the stable DPPH● radical (2,2-diphenyl-1-picrylhydrazyl), following the method by Brand-Williams et al. [58]. Antioxidant activity quantification was expressed as mean ± standard deviation in μM Trolox.g^−1^ extract (antioxidant capacity equivalent to Trolox).

To determine the antioxidant capacity, using the DPPH method, of the essential oils (EO), the radical was prepared by dissolving 3.94 mg of DPPH● (2,2-Diphenyl-1-picrylhydrazyl) (SIGMA ALDRICH^®^, St. Louis, MO, USA) in 100 mL of ethanol. Reaction mixtures were prepared with EO concentrations ranging from 5 to 150 μg mL^−1^ along with the DPPH radical cation. Specifically, 50 μL of EO was mixed with 950 μL of ethanol (MERCK^®^), 2 mL of DPPH radical solution, and then brought to a total volume of 4 mL with ethanol, followed by thorough homogenization. The mixture was allowed to react in the dark for 30 min, and the absorbance of the reaction mixture was measured using a spectrophotometer at 517 nm. The scavenging of the free radical was expressed as a percentage of inhibition (%I) of the DPPH radical activity, where Abs DPPH represented the absorption of the DPPH radical solution and Abs DPPH represented the absorption of the sample [58].

### 3.5. Evaluation of the Antimicrobial Activity of Bio-Oil

The resuspension of the inoculum was performed 24 h prior to seeding on Mueller Hinton Agar (MHA) (HIMEDIA^®^, Modautal, Germany), a non-selective medium with minimal interference. Then, the seeding was incubated in an oven at 36 °C. After this period, in a sterilized tube, 3 to 5 bacterial colonies were inoculated into 5 mL of sterile Mueller Hinton broth (MHB) (HIMEDIA^®^), and then adjusted spectrophotometrically to achieve a concentration of 1 × 10^8^ colony-forming units (CFU)/mL. Once this concentration was reached, it was diluted with MHB to achieve a concentration of 5 × 10^5^ CFU/mL. Samples were diluted with 10% dimethyl sulfoxide (DMSO) and MHB at concentrations of 1000 µg, 500 µg, 250 µg, 125 µg, and 62.5 µg. After inoculum and sample dilution, 100 µL of each was inoculated into a 96-well plate, along with positive and negative controls. This plate was then incubated in an oven for 24 h. Following incubation, the minimum inhibitory concentration (MIC) was determined based on turbidity or using a redox reagent. The minimum bactericidal concentration (MBC) was determined by transferring 10 µL from selected wells and streaking them onto MHA plates by exhaustion. These plates were then incubated in an oven for 24 h at 36 °C, after which CFUs were counted to determine the minimum bactericidal concentration (MBC) [59].

### 3.6. Cell Viability Assay

#### 3.6.1. Ethics Committee

To assess cell viability, venous blood was collected from healthy volunteers who had signed the informed consent form (ICF). This study was approved by the Institutional Review Board for Research Ethics Involving Human Subjects in the Health Sciences sector of UFPA (CEP-ICS/UFPA), under protocol number 3544380 and CAAE (Certificate of Presentation for Ethical Appreciation) number 12776619.0.0000.0018.

#### 3.6.2. Obtaining Peripheral Blood Mononuclear Cells (PBMCs)

Blood samples (5 mL of venous blood) were collected with 5% ethylenediaminetetraacetic acid (EDTA) (New Prov). Subsequently, 5 mL of 0.9% saline solution was added and the mixture was homogenized. The homogenized sample was then transferred to another tube containing 3 mL of Histopaque^®^-1077 (SIGMA ALDRICH^®^, St. Louis, MO, USA). The tube was centrifuged for 15 min at 1500 RPM to separate the mononuclear leukocyte layer. After centrifugation, the mononuclear leukocyte layer was collected and washed with RPMI 1640. The cells were then centrifuged to obtain the cell pellet. The pellet was resuspended in 1 mL of RPMI-1640 supplemented with 1% penicillin/streptomycin and 10% fetal bovine serum for viable cell counting, performed using 0.4% trypan blue staining.

#### 3.6.3. Trypan Blue Exclusion Method and Cellular Morphology Evaluation

For the cell viability assay, 2 × 10^5^ cells were incubated with the fractions at different dilutions for 30 min in a 5% CO_2_ incubator at 37 °C. Subsequently, in a microcentrifuge tube, a 50 µL aliquot was mixed with 0.4% trypan blue at a 1:1 ratio and evaluated using a hemocytometer for cell counting. Stained cells were considered non-viable (blue) and unstained cells viable (translucent). The percentage of viable cells was calculated using the following formula: number of viable cells/total number of cells counted × 100. For assessing cellular morphology, after the incubation period, a 60 µL aliquot was cytocentrifuged and subsequently stained with a panoptic stain (New Prov).

#### 3.6.4. Statistical Analysis

The data were subjected to statistical analysis, where each parameter was initially analyzed for possible outliers using the calculation of the interquartile range. The Student’s *t* test was used to determine the existence of significant differences for each parameter analyzed in each group from the beginning to the end of the study. For each parameter analyzed, a two-way analysis of variance (ANOVA) was performed, followed by the Tukey test for pairwise mean comparisons. Pearson’s correlation test was conducted to assess possible correlations between parameters. Results were considered statistically significant for * *p* ≤ 0.05, ** *p* < 0.01, or *** *p* < 0.00001.

## 4. Conclusions

It is possible to conclude that both the aqueous fraction and the bio-oil demonstrated significant activity against the tested strains, especially at the 1:40 dilution. This indicates that, even at low concentrations, the phenolic and oxygenated compounds present in the waste used in the matrix exhibit biological activity. However, further tests are necessary with strains of different natures and fungal species to broaden the understanding of the effects of these compounds on microorganisms.

The analysis of the DPPH and TEAC methods indicates that antioxidant activity is notably high in the organic fraction (PLO) of açaí, regardless of the tested dilution concentrations. This activity can be attributed to the high content of phenolic compounds found in the organic fraction of the açaí seed.

The research results indicate that lower concentrations of HCl (0.5 M and 1.0 M) at 450 °C cause significant cytotoxicity, reducing the number of cells and altering their morphology. This suggests that temperature and HCl concentration impact the composition of aqueous fractions from açaí pyrolysis. Increased acidity, associated with the hydrolysis of triacylglycerols, increases the production of free fatty acids, impairing the biological properties. Thus, the combination of acid impregnation and high temperatures results in toxic compounds. However, açaí showed promising potential in cytotoxicity tests, allowing for further testing of antioxidant and antimicrobial activity at dilutions with lower cytotoxic effects.

## Figures and Tables

**Table 1:** Chemical composition of bio-oil obtained through the pyrolysis of açaí seeds at 450 °C.

Chemical Compound	Bio-Oil (Área%)
**Linear hydrocarbons**	**9.64**
Undecane	1.12
Tridecane	2.48
Pentadecane	2.29
Dodecane, 5,8-diethyl	1.63
6-tridecene	2.12
**Cyclic and aromatic hydrocarbons**	**11.89**
Cyclohexene, 6-(2-butenylidene)-1,5,5-trimethyl	1.85
Naphthalene	4.40
Naphthalene, 1-methyl	2.39
1H-Indene, 1-ethylidene	3.25
**Carboxylic acids**	**8.53**
Dodecanoicacid	4.31
Tetradecanoicacid	4.22
**Esters**	**4.07**
Undecanoicacid, 10-methyl-, methylester	1.10
Methyltetradecanoate	2.97
**Ketones**	**3.53**
2-Pentanone, 4-hydroxy-4-methyl	1.88
2-Cyclopenten-1-one, 2,3-dimethyl	1.65
**Phenolic compounds**	**55.70**
Phenol	15.93
Methylphenol	20.53
Dimethylphenol	10.09
Methoxyphenol	4.58
Ethylmethoxyphenol	4.57
**Furans**	**5.75**
Benzofuran, 2-methyl	1.88
Furan, 2-(2furanylmethyl)-5-methyl	2.09
Benzofuran, 4,7-dimethyl	1.78
**Aldehydes**	**0.91**
Cinnamaldehyde, β-methyl-	0.91
**Total**	**100**

**Table 2:** Chemical composition of the bio-oil and aqueous phase obtained through the pyrolysis of chemically activated açaí with a 2.0 M KOH solution at 450 °C.

Chemical Compound	Bio-Oil (Area%)	Aqueous Phase (Area%)
**Alcohols**	**10.43**	**26.61**
2,3,4,5,6-Pentamethylbenzyl	1.74	-
2-Furanmethanol	1.58	8.94
Benzenemethanol α-ethyl-4-methoxy Cyclohexanol	1.80	-
5-methyl-2-(1-methylethyl)-, (1α,2α,5β)	3.80	-
2,4-Dimethyl-2-oxazoline-4 methanol	-	17.67
1-Hexadecanol, 2-methyl	1.51	-
**Linear Hydrocarbons**	**12.13**	**3.39**
Decane	1.23	-
Undecane	1.59	-
Tridecane	2.60	-
Tetradecane	2.33	-
7-Tetradecane	2.86	3.39
Nonadecane	1.52	-
**Cyclic and aromatic hydrocarbons**	**13.59**	**-**
Bicyclo[4.2.0]octa-1,3,5-triene	3.13	-
Ethylbenzene	2.21	-
Toluene	1.95	-
1,2,4,4 Tetramethylcyclopentene	1.30	-
1,3-Cyclopentadiene, 5-(1-methylpropylidene	0.89	-
Cyclohexane	3.25	-
Cyclohexane, 1,2,4-tris(methylene)	0.86	-
**Nitrogenated compounds**	**4.38**	**13.05**
4-(2,5-Dihydro-3 methoxyphenyl)butylamine	2.44	-
Tricyclo[3.1.0.0(2,4)] hex-3-ene 3-carbonitrile	1.94	-
N-Tert.-butyl-N-(2 propenyl)amine	-	6.05
2-Propen-1-amine, N,N-bis(1-methylethyl) Aziridine	-	3.62
2-(1,1-dimethylethyl)-1-ethyl 3-methyl-,trans-	-	3.38
**Carboxylic acids**	**0.97**	**9.23**
Butanoicacid,4-hydroxy	0.97	-
Butanedioic acid, methylene	-	2.49
Butanoicacid,4-hydroxy	-	6.74
**Esters**	**1.29**	**3.32**
Acetic acid, 7-hydroxy 1,3,4,5,6,7-hexahydro-2H naphthalen-4a-ylmethyl ester	1.29	-
Carbamicacid, phenyl ester	-	3.32
**Ketones**	**7.07**	**44.38**
2-Cyclopenten-1-one, 2,3-dimethyl	1.86	-
2-Cyclopenten-1-one, 2-methyl	0.90	-
4-(3,7,7-Trimethyl-2 oxabicyclo[3.2.0]hept-3-en-1 yl)but-3-en-2-one	3.02	-
Spiro[2.3]hexan-5-one, 4,4-diethyl	1.29	-
2-Pentanone, 4-amino-4-methyl	-	32.54
2-Propanone, (1 methylethylidene)hydrazone	-	2.29
4-Piperidinone, 2,2,6,6-tetramethy	-	9.55
**Phenolic compounds**	**42.98**	**-**
Phenol	5.73	-
Methyl phenol	5.60	-
Dimethyl phenol	9.74	-
Trimethyl phenol	8.26	-
Ethyl methyl phenol	4.84	-
Dimethoxy phenol	2.90	-
Ethyl phenol	4.316	-
Ethyl methoxy phenol	2.94	-
**Non-identified fraction**	**5.81**	**-**
**Total**	**100**	**100**

**Table 3:** Chemical composition of the compounds obtained by the pyrolysis of açaí (*E. oleracea*).

Chemical Composition Ci (Area%)	2.0 M KOH		
	350 °C	400 °C	450 °C
Alcohols	2.34	20.74	26.62
Carboxylic Acids	4.05	15.02	9.23
Ketones	52.81	44.38	19.69
Oxygenates	40.80	19.86	44.46
$\sum_{i}^{n}Ci$	100.00	100.00	100.00
Acidity (mg KOH/g)	118.9	26.8	17.9

**Table 4:** Antioxidant capacity of bio-oils obtained by the pyrolysis of açaí seeds (*E. oleracea*) by the TEAC method.

TEAC (mmol/L)		
Dilution	2.0 M KOH	
	350 °C	400 °C
1:10	2.372	2.372
1:20	2.342	2.28
1:40	2.285	2.226
1:80	2.022	1.375
1:160	1.584	1.019
1:320	1.18	0.239

**Table 5:** Antioxidant capacity of bio-oils obtained from the pyrolysis of açaí seeds (*E. oleracea*) chemically activated with 2.0 M HCl solutions at temperatures of 350, 400, and 450 °C on a laboratory scale.

TEAC (mmol/L)			
Dilution	2.0 M HCl		
	350 °C	400 °C	450 °C
1:10	2.34	2.32	2.31
1:20	2.25	2.23	2.08
1:40	1.22	1.19	1.15
1:80	0.96	0.94	0.90
1:160	0.93	0.90	0.87

**Table 6:** Antioxidant capacity of bio-oils obtained by the pyrolysis of açaí seeds (*E. oleracea*) chemically activated with 0.5 M and 1.0 M KOH solutions at temperatures of 450 °C on a laboratory scale.

TEAC (mmol/L)		
Dilution	KOH 450 °C	
	0.5 M	1.0 M
1:10	2.357	2.292
1:20	2.272	2.28
1:40	2.203	2.252
1:80	2.163	1.513
1:160	1.664	0.885
1:320	0.56	0.57

**Table 7:** Antioxidant capacity of bio-oils obtained by the pyrolysis of açaí seeds (*E. oleracea*), using different molarities (KOH solution of 0.5, 1.0, and 2.0 M), at 450 °C.

TEAC (mmol/L)			
Dilution	KOH 450 °C		
	0.5 M	1.0 M	2.0 M
1:10	2.45	2.45	2.28
1:20	2.45	2.45	2.03
1:40	2.39	2.39	1.19
1:80	2.36	2.36	1.12
1:160	0.93	0.90	0.87
1:320	1.60	1.59	0.56
1:640	0.97	0.97	0.47

**Table 8:** Antioxidant capacity of the aqueous phase of bio-oils obtained by the pyrolysis of Açaí seeds (*E. oleracea*), at different temperatures (350 °C and 400 °C), chemically activated with 2.0 M KOH on a laboratory scale.

DPPH (µM/L)		
Dilution	2.0 M KOH	
	350 °C	400 °C
1:10	2.372	2.372
1:20	2.342	2.28
1:40	2.285	2.226
1:80	2.022	1.375
1:160	1.584	1.019

**Table 9:** Antioxidant capacity of the organic fraction of bio-oils obtained by the pyrolysis of açaí seeds (*E. oleracea*), at different temperatures (350 °C and 400 °C), chemically activated with 2.0 M KOH on a laboratory scale.

DPPH (µM/L)		
Dilution	2.0 M KOH	
	350° C	400 °C
1:20	2.145	1.805
1:40	1.701	2.271
1:80	1.748	2.675
1:160	2.170	3.248
1:320	2.864	3.315
1:640	2.886	3.121

**Table 10:** Antioxidant capacity of the aqueous fraction of bio-oils obtained by the pyrolysis of açaí seeds (*E. oleracea*), chemically activated with 2.0 M KOH at different molarities (0.5 M–2.0 M) at 450 °C on a laboratory scale.

DPPH (µM/L)			
Dilution	KOH 450 °C		
	0.5 M	1.0 M	2.0 M
1:10	0.206	0.256	0.714
1:20	0.199	0.278	1.111
1:40	0.365	0.362	1.694
1:80	0.402	0.583	2.618
1:160	0.893	1.185	2.598

**Table 11:** Antioxidant capacity of the aqueous fraction of bio-oils obtained by the pyrolysis of açaí seeds (*E. oleracea*), chemically activated with 2.0 M KOH at different molarities (0.5 M–2.0 M) at 450 °C on a laboratory scale.

DPPH (µM/L)		
Dilution	KOH 450 °C	
	0.5 M	1.0 M
1:20	0.672	0.712
1:40	1.475	1.495
1:80	1.277	2.244
1:160	2.053	2.869
1:320	2.913	3.035
1:640	2.722	3.518

**Table 12:** Minimum inhibitory and bactericidal concentrations of wash water from the pyrolysis of açaí seed impregnated with KOH against *E. coli* strain.

Nature	Pretreatment	Temperature	Code	MIC	MBC
Wash water	KOH (0.5 M)	-	L1	>1:10	>1:10
Wash water	KOH (1.0 M)	-	L2	1:40	1:40
Wash water	KOH (2.0 M)	-	L3	1:40	1:40

**Table 13:** Minimum inhibitory concentrations and bactericidal concentrations of the aqueous fraction of bio-oil from the pyrolysis of açaí seeds impregnated with KOH and HCl against *E. coli* strain.

Nature	Pretreatment	Temperature	Code	MIC	MBC
Aqueous Phase	KOH (0.5 M)	450 °C	HM1	>1:10	>1:10
Aqueous Phase	KOH (1.0 M)	450 °C	HM2	1:20	1:20
Aqueous Phase	KOH (2.0 M)	450 °C	HC3	1;40	1:40
Aqueous Phase	HCL (0.5 M)	450 °C	HM1	1:40	1:40
Aqueous Phase	HCL (1.0 M)	450 °C	HM2	1:40	1:40
Aqueous Phase	HCL (2.0 M)	450 °C	HC3	>1:40	>1:40

**Table 14:** Minimum inhibitory concentrations and bactericidal concentrations of the organic fraction of bio-oil from the pyrolysis of açaí seeds impregnated with KOH against *E. coli* strain.

Nature	Pretreatment	Temperature	Code	MIC	MBC
Organic Phase	KOH (0.5 M)	450 °C	PM1	1:40	1:40
Organic Phase	KOH (1.0 M)	450 °C	PM2	1:80	1:80
Organic Phase	KOH (2.0 M)	350 °C	PT1	>1:40	>1:40
Organic Phase	KOH (2.0 M)	400 °C	PT2	1:80	1:80

**Table 15:** Minimum inhibitory and bactericidal concentrations of wash water from açaí seed pyrolysis impregnated with KOH against the *S. aureus* strain.

Nature	Pretreatment	Temperature	Code	MIC	MBC
Washing water	KOH (0.5 M)	-	L1	1:5	1:20
Washing water	KOH (1.0 M)	-	L2	>1:10	1:40
Washing water	KOH(2.0 M)	-	L3	>1:10	1:40

**Table 16:** Minimum inhibitory and bactericidal concentrations of the aqueous fraction of bio-oil from açaí seed pyrolysis impregnated with KOH against the strain of *S. aureus*.

Nature	Pretreatment	Temperature	Code	MIC	MBC
Aqueous Phase	KOH (2.0 M)	350 °C	C.N/A	1:40	1:40
Aqueous Phase	KOH (2.0 M)	400 °C	C.B	>1:40	>1:40
Aqueous Phase	KOH (2.0 M)	450 °C	C.A	>1:40	>1:40

**Table 17:** Minimum inhibitory and bactericidal concentrations of the organic fraction of bio-oil from açaí seed pyrolysis impregnated with HCL against the strain of *S. aureus*.

Nature	Pretreatment	Temperature	Code	MIC	MBC
Organic Phase	HCL (2.0 M)	400 °C	PLO/B	1:5	1:40
Organic Phase	HCL (2.0 M)	450 °C	PLO/A	>1:5	>1:40

## Data Availability

The datasets generated for this study are available on request to the corresponding author.
